# Incorporating Anthropogenic Influences into Fire Probability Models: Effects of Human Activity and Climate Change on Fire Activity in California

**DOI:** 10.1371/journal.pone.0153589

**Published:** 2016-04-28

**Authors:** Michael L. Mann, Enric Batllori, Max A. Moritz, Eric K. Waller, Peter Berck, Alan L. Flint, Lorraine E. Flint, Emmalee Dolfi

**Affiliations:** 1 Department of Geography, The George Washington University, Washington, DC, United States of America; 2 Department of Environmental Science, Policy, and Management, University of California Berkeley, Berkeley, CA, United States of America; 3 CEMFOR—CTFC, InForest Joint Research Unit, CSIC-CTFC-CREAF, Solsona, E-25280, Spain; 4 CREAF, Cerdanyola del Vallès, E-08193, Spain; 5 U.S. Geological Survey, Western Geographic Science Center, Menlo Park, CA, United States of America; 6 Department of Agriculture and Resource Economics, University of California Berkeley, Berkeley, CA, United States of America; 7 US Geological Survey, California Water Science Center, Sacramento, CA, United States of America; University of Nevada Reno, UNITED STATES

## Abstract

The costly interactions between humans and wildfires throughout California demonstrate the need to understand the relationships between them, especially in the face of a changing climate and expanding human communities. Although a number of statistical and process-based wildfire models exist for California, there is enormous uncertainty about the location and number of future fires, with previously published estimates of increases ranging from nine to fifty-three percent by the end of the century. Our goal is to assess the role of climate and anthropogenic influences on the state’s fire regimes from 1975 to 2050. We develop an empirical model that integrates estimates of biophysical indicators relevant to plant communities and anthropogenic influences at each forecast time step. Historically, we find that anthropogenic influences account for up to fifty percent of explanatory power in the model. We also find that the total area burned is likely to increase, with burned area expected to increase by 2.2 and 5.0 percent by 2050 under climatic bookends (PCM and GFDL climate models, respectively). Our two climate models show considerable agreement, but due to potential shifts in rainfall patterns, substantial uncertainty remains for the semiarid inland deserts and coastal areas of the south. Given the strength of human-related variables in some regions, however, it is clear that comprehensive projections of future fire activity should include both anthropogenic and biophysical influences. Previous findings of substantially increased numbers of fires and burned area for California may be tied to omitted variable bias from the exclusion of human influences. The omission of anthropogenic variables in our model would overstate the importance of climatic ones by at least 24%. As such, the failure to include anthropogenic effects in many models likely overstates the response of wildfire to climatic change.

## Introduction

Although climate and weather are key drivers of wildfire occurrence across California, humans are currently responsible for igniting approximately 95% of wildfires in the state [1]. Drawn by both natural and suburban amenities, Californians have built in or near areas of natural vegetation, known as the wildland-urban interface (WUI), much of which is fire-prone [2,3]. For instance, in San Diego County three of every four homes were built within such risk-prone WUI areas [1]. Consequently, in State Responsibility Areas (SRA), where the state responds to fire activity, an average of $160.3 million dollars in fire-related damage to structures was reported annually between 1999 and 2011, with a total of 12,682 structures destroyed [4]. During this same time period, California and the U.S. Forest Service reported spending more than $5.18 billion on wildfire suppression in the state [4,5]. In SRA lands annual costs between 2012 and 2014 have risen to $230 million annually [6]. These figures highlight the importance of interactions between humans, natural systems, and disturbances such as wildfire. Actors at local, state, and federal levels should incorporate an understanding of controls on fire activity and inherent uncertainties, given that people and wildfires must ultimately coexist [7].

A number of statistical and process-based wildfire models exist for California [8–12], yet our ability to predict the location and number of future fires is limited. There is general consensus that fire occurrence will increase with climate change, with the exception of very dry scenarios [13], but projections for the number of wildfire events by the end the century vary dramatically (e.g., +9 to 13% [11], +40 to 49% [14], and +34 to 53% [12]). Similarly, the total area burned in the state is estimated to increase from somewhere between +15 and 50% [10,15]. Much of this inter-model variation can be explained by differences between the scenarios of change, the climate models, or the assumptions used in such simulations (e.g., effects of climate changes on plant communities, and the omission or inclusion of anthropogenic effects).

Ongoing expansion of human communities into fire-prone habitats is a primary source of model uncertainties and inter-model variation that needs to be further explored. Relationships between fire and global population patterns have been identified [16–18], and in coarse resolution models anthropogenic effects are assumed to manifest primarily through the availability of ignitions [19]. Relatively little is known, however, about influences of fire suppression, land use patterns, and other human activities at finer scales of analysis [1,9,20]. Empirical work in California [1] supports the proposed relationship between fire and human activity and the “pyric transition”–that is, the observation that fire activity initially increases in parallel with increased human influence, but then decreases beyond some point as characteristics of the built urban environment and increased suppression effort reduce it [21,22].

**Fig 1:**
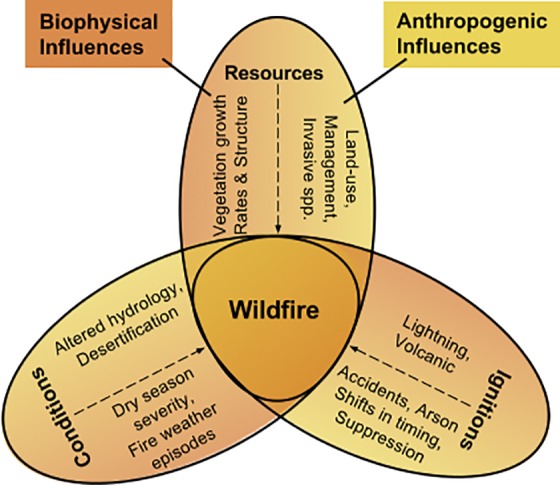
Biophysical and anthropogenic determinants of wildfire. Interactions of wildfire requirements as regulated by biophysical and anthropogenic influences. Anthropogenic determinants are shaded in yellow and biophysical influences in orange.

In the work presented here, we further develop the model presented in Krawchuk and Moritz [9] to explicitly consider how humans might impact components of the “fire regime triangle” (Fig 1) [8,19,23,24]. A key contribution is much improved handling of the effects of housing development and land tenure, the influence of which is largely unknown. In this framework, wildfire is regarded as an abiotic ecological process that, like its biotic counterparts (e.g., vegetation), has certain habitat requirements (i.e., conditions suitable for combustion, fuel, and an ignition source), the variation of which constrains its occurrence. In this study we use five different model specifications (including and excluding fire occurrence constraints) to test specific hypotheses about the role of anthropogenic and climatic influences on wildfire over California. Specifically, we 1) test whether the inclusion of housing density and proximity to populated places improves model performance compared to ‘natural drivers only’ models, 2) test if inter-annual variability in water availability explains fire patterns unexplained by included human and natural determinants, and 3) whether public land management and human behaviors significantly influence fire activity. Then we use the best model specification to assess California’s wildfire activity (fire count and burned area) by 2050 under two specifications of climate change that depict different futures in terms of temperature and precipitation patterns over the state.

## Methods

### Model overview

We develop a model for the state of California to estimate fire counts and burned area for the 1951–2050 period using a zero-inflated negative binomial regression. We use spatially explicit data at a 1080m spatial resolution summarized in 25-year time periods. As such, the dependent variable, fire counts, is rasterized from fire perimeters while independent variables are representative of the mean or standard deviation of climatic conditions and anthropogenic influence within this period. These spatial and temporal resolutions can be considered representative of landscape scale fire regimes for California [8,9]. 1080m is chosen rather than 1000m because it is a multiple of eco-hydrological data that plays a critical role in this research. Regressions on the 1976–2000 period are used to calibrate the model, and validated by comparing actual and modeled burn counts for the 1951–1975 period. Finally, forecast estimates for the 2001–2025 and 2026–2050 periods are developed under the CMIP3 Parallel Climate Model (PCM) and the Geophysical Fluid Dynamics Laboratory (GFDL) climate models under the A2 emissions scenario. This approach aims to bound uncertainty of climate outcomes on fire activity [9,25]. Our model builds on the framework developed by Krawchuk and Moritz [9] by better integrating major constraints of fire activity (Fig 1) including climate, drivers of vegetation patterns, and models forecasting the location of human settlements and activity. We also improve the selection of estimator type, test for the use of zero-inflation, and attempt to create a set of variables more easily compared with established relationships between climate, human and natural systems, and wildfire frequency.

In this modeling exercise, we incorporate determinants of vegetation patterns by explicitly integrating patterns of eco-hydrologic variables relevant to plant productivity and water availability. Additionally, anthropogenic ignition and suppression patterns are modeled with data on intensity of human settlements and activities, while attempting to capture differences in land management based on private and public ownership.

The dependent variable is measured as the number of wildfires from 1976–2000 for each pixel. Wildfire perimeter data was collected from the California Fire Resource and Assessment Program [26]. Despite some shortcomings this data is considered the most complete record of fire perimeters for the state, compiling information from CALFIRE/FRAP, United States Department of Agriculture, Bureau of Land Management, the National Park Service, and Bureau of Indian Affairs and Department of Defense [27]. Generally, USFS wildland fires of 10 acres or greater and CALFIRE fires of 300 acres are included in the database. Our 1080m resolution equates to 288 acres, roughly matching the CALFIRE minimum fire size. Due to the historical nature of this data, records are less complete and more heavily generalized in earlier periods. This includes the simplification of complex spatial boundaries. Fire perimeters were converted to raster format by rasterizing based on centroids at 270m resolution and then aggregated to 1080m using a weighted sum, providing the total number of fires including fractional fires. These sums are then rounded to the nearest whole number, as is required for count distribution modeling. The use of pixels here implies that our fire count estimates are based on the frequency at which an entire cell would be expected to burn. For more detail on the use of raster data for wildfire modeling see Krawchuck and Moritz [9].

We considered a set of 10 independent variables (Table 1) from diverse sources. Biophysical climate and hydrologic data are provided by the California Basin Characterization Model (BCM) [25–27] and are derived for historical periods from PRISM gridded climate data [28] and for future periods from Coupled Model Intercomparison Project Phase 3 (CMIP3) for PCM and GFDL A2. Climate inputs are spatially downscaled following [29] and used to drive the water balance model to calculate hydrologic variables AET and CWD. The spatial downscaling is shown to have no adverse impacts on the estimates of climate, improving the estimates in many cases [29]. Limitations of the hydrologic calculations of the BCM are discussed at length in [30]. Historical and forecast housing densities are provided by Mann el al. [28], data on elevation and slope is derived from Shuttle Radar Topography Mission [29], the location of campsites is web-scraped from the Reserve America System [30], public land extent is obtained from the multisource land ownership database [31], and a ten year (2001–2011) representative period of lightning strike intensity data (strikes/km^2^/year) are provided by Weather Services International [32]. Lightning strike data are treated as time invariant and used for all time periods, assuming thus they are representative of the spatial distribution of strikes. All data is restricted to the extent of California’s state boundary, while omitting all inland water bodies and croplands as defined by [33]. All other data without meaningful historical records and forecasts are treated as time invariant (see Table 1).

**Table 1:** Description of the 10 variables considered to estimate fire occurrence and burned area.

Variable	Variable Name	Description (Units)	Time Variant
Wildfire count	*Wildfire*	Number of wildfires in a 25 year period (count)	True
**Natural**			
Annual mean actual evapotranspiration	*aetAave*	Average annual evapotranspiration (mm)	True
Annual mean climatic water deficit	*cwdAave*	Average annual deficit (mm)	True
Annual std. dev. climatic water deficit	*cwdAsd*	Annual standard deviation in climatic water deficit (mm)	True
Average lightning strike density	*Lightning*	Lightning strike intensity data 2001–2011 (Strikes/Km^2^/Year)	False
**Terrain**			
Slope	*Slope*	Terrain slope (°)	False
Elevation	*Elev*	Terrain elevation (m)	False
**Human**			
Maximum housing density	*DenMax25km*	Maximum density in a 25 cell radius circle (units / acre)	True
Distance to populated places	*Ppall*	Euclidean distance to census populated places (m)	False
Distance to camp grounds	*CampDist*	Euclidean distance to public and private campgrounds (m)	False
Public land	*PublicLand*	Public land ownership dummy	False

We use 5 different model specifications by including and excluding some of the constraints on fire activity in order to: test the significance of natural versus human drivers of wildfire variability (Models 1 and 2), evaluate the importance of including the inter-annual variability of water availability on model performance (Model 3), assess the effects of other human influences aside from housing density, specifically distance to nearest campsite (*CampDist*) and a public land binary variable (*PublicLand*) (Models 4 and 5, respectively).

### Climate futures

Uncertainty in projecting future climates is incorporated by using two future climate projections, the CMIP3 PCM and GFDL climate models, under the A2 emissions scenario. Although CMIP5 data has recently become available, downscaled eco-hydrological data required for this study were not available at the time of model development and analysis. Since CMIP5 projections are an incremental advance over CMIP3 data [34–36], this does not limit the relevance of our analysis as a tool for assessing anthropogenic influences on future fire activity. Furthermore, CMIP3, GFDL and PCM models are still relevant and useful because these data are: a) well vetted representations of California’s future climate, and have been demonstrated to replicate historical temperatures and interannual sea surface temperatures that drive California’s precipitation regime [37], b) adopted by California’s agencies [9,38], c) representative of California’s climate bookends, d) are used broadly in other scientific literature [39–41], and e) facilitates comparison to findings from a variety of other study using similar projections [9,11,12,15,19].

In simplified terms, the GFDL climate by midcentury can be characterized as having a wetter south and drier north. The PCM climate mirrors this as it predicts a drier south and wetter north. The GFDL model experiences less precipitation during the 2000–2025 period, with the exception of the mountain ranges of northwestern California. In 2026–2050, this pattern reverses with an increase in precipitation across much of the southern portion of the Central Valley, and the southwestern and southeastern coastal and semi-arid zones, respectively. On the other hand, for the 2001–2025 period in the PCM model low to mid elevation zones of the southeastern deserts experience increasing water availability, while in 2026–2050 water availability declines throughout the south and increases above 37° latitude.

### Anthropogenic influences

We incorporate anthropogenic ignition and suppression patterns by modelling the extent and density of human settlements throughout the state. That is, because ignition data are limited, we use two variables correlated with ignitions (distance to population centers, housing density) as proxies [1]. These variables also likely correlate with demand for firefighting services and suppression effort. Given that human activity is a dynamic process, historic estimates and forecasts of housing density are integrated in each time step [28]. The use of time variant housing density data may allow the model to better simulate the net effects of human ignitions and suppression over time. Land management and human activities in rural and wildland areas are also integrated through land ownership variables (privately and publicly held land), and the distance to public campsites.

### Vegetation amount and flammability

**Fig 2:**
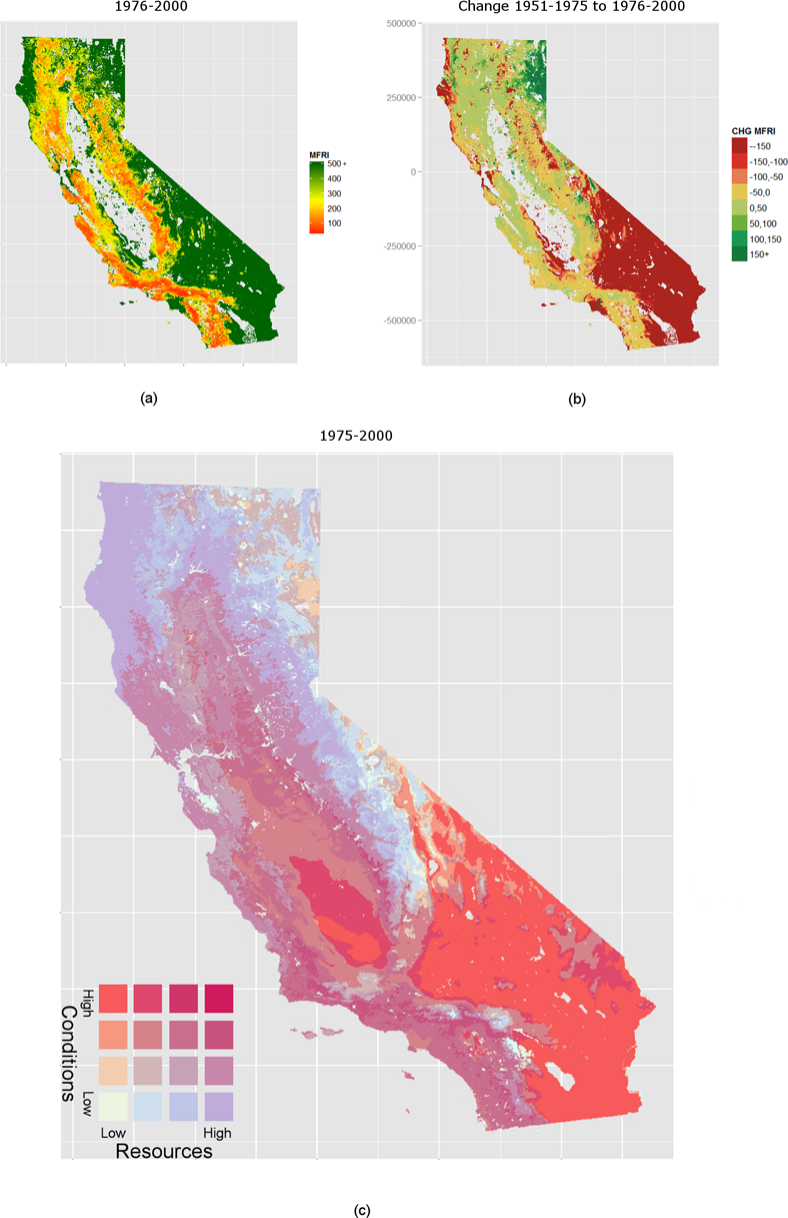
Mean fire return interval (mFRI) estimate 1976–2000. Mean fire return interval (mFRI) estimate 1976–2000 (A), and its change from the historical period (1951–1975) (B). In (a) low mFRIs (red) designate short intervals between fire events, and high mFRI designate long intervals (green). In (B) positive values (green) indicate an increase in mFRI (i.e., decrease in the number of fires) between the estimation and previous period (1976–2000 less 1951–1975), whereas negative values (red) indicate a decreasing mFRI or an increase in the number of fires. (C) Varying constraint map of fire resource and conditions 1976–2000: Four categories of fire resources as proxied by AET, and fire conditions proxied by CWD. Color is determined by the unique combinations of resources and conditions. Where increasingly favorable fire conditions are indicated with deepening red hues, and increasingly favorable resource classes are indicated with deeper hues of blue.

The distribution of vegetation types is largely controlled by the biophysical effects of climatic variables [42–44]. Actual evapotranspiration (AET) is recognized as a robust estimate of vascular plant productivity [45] and can be regarded as a proxy of biomass accumulation and regrowth. Climatic water deficit (CWD) is the evaporative demand exceeding available soil moisture [45] and can be seen as proxy for conditions favorable for vegetation flammability and wildfire occurrence. These two eco-hydrological variables integrate complex interactions of heat, water supply and demand, terrain, and edaphic properties into simple measures of seasonal weather on plant community structure and productivity, as well as water stress and fire season severity [9,46–49]. As such, AET and CWD are good indicators of potential changes in the distribution of fuel amount and its flammability (i.e., two of the three biophysical categories shown in Fig 1), under changing climates. California’s diverse ecosystems and wildfire habitats can thus be roughly identified through the interactions of these proxies for flammability conditions and resources to burn (Fig 2C).

### Model Outputs

We convert estimates of fire counts per pixel to estimates of mean fire return interval (mFRI) to facilitate model interpretation. mFRI is defined as the expected number of years between fire events for any given fire regime. Therefore, for any given twenty-five-year time period (e.g. 1976–2000), mFRI is calculated as twenty-five divided by the estimated number fires. Burned area is estimated as the number of times an entire cell burns in a twenty-five-year period multiplied by the area of that cell.

We can visually examine the contributions of certain sets of variables to the total fire count by finding the difference between the number of fires with and without a set of variables fixed at their global mean value. The global mean corresponds to the mean value of all cell values across California for a particular set of data. For instance, we can estimate the ‘human’ contributions to the fire count by finding the difference between fire counts estimated from a full model incorporating both ‘natural’ and ‘human’ variables, and fire counts estimated with the latter (*DenMax25km*, *Ppall*, and *CampDist*, *i*.*e*. *‘human’ variables*) held at their global mean. The same can be done with ‘natural’ variables (*aetAave*, *cwdAave*, *Lightning*, *elev* and *slope*) to estimate their contribution to the total fire count.

### Statistical Approach

The techniques required for this study are complicated by two key factors. First, the dependent variable holds the total number of wildfires for a particular time period. This ‘count’ data is often modeled with the use of Poisson and negative binomial distributions. Second, wildfire data can be thought of as ‘presence only’ data, whereby researchers only record the presence of a fire, and usually only if they meet certain requirements (such as a minimum size). This data generation process can be simulated with the use of zero-inflated models. These principals are not necessarily straightforward and also apply to many different spatial distribution modeling problems; we therefore provide the following section to help clarify these definitions and describe our approach to handling them.

### Modeling Background

‘Presence only’ data is commonly found in ecological studies [50]. Conceptually we can think of the example of bird habitat studies, where ecologists will typically report only the presence of a bird when it is detectable at a particular time and place. That bird’s absence from another similar site may have little do with its suitability as habitat, but instead reflects the fact that birds must choose from a variety of suitable sites at any given time. Additionally, the failure to observe a particular species of bird does not imply that it was not actually present, but instead may have been difficult to detect or the observation period was too short. Similarly, fire perimeter data can be treated as ‘presence only’ for two reasons: 1) wildfire data is only recorded for observed fires and if they reach a certain threshold for size, and 2) ideal circumstances do not always lead to the presence of wildfire within a given observation period. This implies that fires may be present but do not reach the recording-threshold due to early suppression or failure to spread (fragmented fuels, varying microclimate conditions), or be present but go unrecorded due to observation failure (due to inaccessibility, poor viewshed). Particularly earlier in the period of record. Alternatively, many areas regularly experience ideal conditions for wildfire but lack an essential ingredient such as an ignition source, or spatially contiguous fuels. Therefore many areas without fire over a given period cannot be considered unsuitable or unlikely to burn, but instead may be primed for wildfire under slightly different circumstances when the key factors align [24]. Following this rationale, wildfire data can be classified in three ways: present and recorded fires (true 1s), present yet unrecorded fires (false 0s), ideal fire-conducive conditions without fire or unrecorded actual fires (false 0s), and non-present wildfires as a result of non-suitable conditions (true 0s). Additional, ‘0s’ are therefore introduced as result of the data generation process. As such, this type of data suffers from ‘excessive zeros’ and must be handled with a particular suite of models, specifically zero-inflated approaches.

Further complicating things, fire data over broader temporal scales can be thought of as ‘count’ data, because any particular location is capable of burning in more than one discrete event. This is most applicable when the resolution of grid cells being modeled is roughly consistent with the finest spatial resolution of the fire observations themselves. For instance, in this study a handful of cells burned 4 times in a 25-year period. Ignoring this could understate the persistent nature of wildfires in some landscapes. Therefore, we use a zero-inflated negative binomial (ZINB) regression, which is capable of handling both count data and excessive zeros. This model estimates the effect of climate, vegetation, and human influences on the total fire count for the 1976–2000 period. Tests to determine the appropriate estimation method can be found in the Specification Tests section below, and coefficient estimates of each independent variable are reported in the results section. For a detailed review of ZINB models please refer to the Supporting Information S1 File (Zero Inflated Negative Binomial Models section).

ZINB regressions model count and zero-inflation as two regressions because the processes generating the count data are different from the processes generating excess zeros. Therefore, the two regressions presented below should be interpreted separately. The first regression (count process) should be interpreted as a one unit change in the independent variable corresponding to a $\hat{\beta }$ unit change in the log count of fires, where $\hat{\beta }$ is an estimated coefficient. The second component of the regression (excess zero) takes the form of a logistic regression, where a one unit change in the independent variable corresponds to a $\hat{\beta }$ unit change in the log odds of being an excessive zero (false zero). In this way the interpretation is the reverse of traditional logistic regression, here positive coefficients correspond to greater odds of that observation being an excess zero.

### Pre-Estimation Tests, Specification, and Model Performance

Before estimating the regression between the dependent (fire counts) and independent (natural and human fire constraints) variables, we must confirm that the estimator uses the correct distribution (Negative Binomial), and that zero-inflation improves the handling of our data. First, to choose between the use of Poisson and Negative Binomial distributions, we test whether a dispersion parameter improves the model using a likelihood ratio test. We find that we can reject the null hypothesis that the data are more likely described by the Poisson distribution (p<0.01), strongly supporting the use of Negative Binomial regression. Second, to choose between zero-inflated and non-inflated negative binomial regression, we apply the Vuong closeness test [51]. We find that we can reject the null hypothesis that the two models are equally close to the actual model in favor of zero-inflation, which is a superior model (p<0.01) [52]. Finally, we also test model performance against the null model (intercepts only) and reject equality of the models (p<0.01) in favor of the full model.

Finally, to identify which independent variables should and should not be included in the final model, we apply the chi-squared test on the difference of log likelihoods, which tests for significant model improvements from the inclusion of additional variables. We evaluate model performance using three measures of out-of-sample prediction error: a) the difference between actual and predicted total km^2^ squared for time *t* = 1976–2000 [*Area km*^*2*^ *Diff (t)*], b) the difference between actual and predicted total km^2^ squared for time *t*-1 = 1951–1975 [*Area km*^*2*^ *Diff (t-1)*], and c) the sum of squared *t* and *t*-1 difference in area.

### Sampling, Spatial Autocorrelation, and Estimation

We apply regression ‘bagging’ techniques to minimize the effect of spatial autocorrelation in model residuals, and to provide more generalizable results, while maintaining an effective sample size for hypothesis testing [9]. We estimate each regression on 30 subsamples using a sampling fraction of 2.5% of all observations (N = 7332). Regression coefficients and standard errors from each of the subsamples are then averaged. This ensures that models are not fit to a particular observed realization of fire events, but instead fit a variety of samples of the population. We also report the number of times that each variable is significant across the 30 subsamples (p<0.10). We use a sparse sampling fraction to ensure that each observation contributes relatively independent information, thereby minimizing the effects of spatial autocorrelation. Without proper treatment, spatial autocorrelation in the residual leads to the underestimation of standard errors, and therefore biases hypothesis testing.

## Results & Outputs

### Anthropogenic and Climatic Influences on Wildfire

Modeling results from the number of wildfire events and its determinants are presented in Table 2. Across model specifications, non-linear relationships between fire and each predicting variable were established through both theory and indicators of model fit. The following section outlines the determination of the final model. The climate only specification (Model 1) does a reasonable job in estimating fire area over California (Table 2), but the inclusion of human drivers of wildfire (Model 2) significantly improves model performance for all statistical indicators (AIC, Log-Likelihood) as well as prediction error for the 1951–1975 and 1976–2000 periods (Table 2). Most measures of model performance improve through the addition of inter-annual variability in CWD, which incorporates fluctuations in conditions favorable to fire occurrence (AIC and log-likelihood in Table 2, Model 3) but improvements are not statistically meaningful (Table 3, Model 1 vs 2). These same results were also true for the suite of possible indicators of variability for AET, precipitation, and maximum temperature (*results not shown*). On the other hand, the difference of log likelihood tests and indicators of prediction error suggest that the inclusion of land ownership, a proxy for public vs. private management practices (*PublicLand*, Model 4), and of human activity in wildlands (*CampDist*, Model 5), significantly improve the model. Therefore, we retain Model 5, which incorporates all significant anthropogenic and natural determinants, to estimate fire counts and area burned, and to assess their alteration under future climates.

**Table 2:** Results from Zero Inflated Negative Binomial Model (Y = Wildfire Count).

	Model 1	Model 2	Model 3	Model 4	Model 5
**Count**					
Intercept	-10.22***	-8.84***	-8.81***	-9.75***	-9.22***
	[30]	[30]	[30]	[30]	[30]
cwdAave	5.88***	4.34***	7.33***	4.80***	4.83***
	[30]	[30]	[30]	[30]	[30]
cwdAave^2^	-2.63***	-1.83**	-3.09***	-1.93**	-1.96**
	[29]	[30]	[30]	[29]	[28]
aetAave	7.37***	6.19***	6.43***	6.59***	6.12***
	[30]	[30]	[30]	[30]	[30]
aetAave^2^	-2.60***	-2.21***	-2.23***	-2.28***	-2.14***
	[30]	[30]	[30]	[30]	[30]
cwdAsd			-1.87^+^		
			[24]		
Log(slope)	0.58***	0.52***	0.47***	0.40***	0.38***
	[30]	[30]	[30]	[30]	[30]
DenMax25km		0.19***	0.16**	0.20***	0.18**
		[30]	[26]	[30]	[30]
DenMax25km^2^		-0.02**	-0.02**	-0.02***	-0.02**
		[29]	[26]	[30]	[29]
CampDist					-0.31**
					[27]
PublicLand				0.54***	0.51***
				[30]	[30]
Theta	1.05^+^	2.41	2.57^+^	3.23	2.63^+^
	[23]	[24]	[26]	[26]	[26]
**Zero-Inflation**					
Intercept	-2.66	-2.72^+^	-2.82	-3.50**	-3.72^+^
	[6]	[19]	[19]	[25]	[23]
Ppall		-5.76**	-6.56**	-6.92**	-7.65**
		[29]	[29]	[29]	[28]
Ppall^2^		2.16**	2.43**	2.65**	2.98**
		[30]	[30]	[30]	[28]
Log(Elev)	-5.95**	-7.56***	-7.62***	-8.93***	-9.56**
	[27]	[30]	[30]	[30]	[29]
Lightning	-11.09^+^	2.84***	3.45***	4.02**	3.57**
	[27]	[30]	[30]	[30]	[29]
**Model Fit**					
AIC	3951.654	3934.364	3933.422	3881.868	3879.41
Log-Likelihood	-1965.83	-1953.18	-1951.71	-1925.93	-1923.71
Area km^2^ Diff (t)	-694	703	3,400	443	1,372
Area km^2^Diff (t-1)	-6,373	-4,658	-1,883	-4,879	-4,017
Sum (Area Diff^2^)	4.11e7	2.22e7	1.51e7	2.40e7	1.80e7

Mean Significance Level

*** p<0.01

** p<0.05

^+^p<0.1 Number of Runs Significant: [#] at p<0.10

Akaike Information Criterion: AIC Actual Area Burned less Predicted: Area Diff by Time Period, where *t* corresponds to the estimation period (1976–2000) and *t-1* to previous period (1951–1975).

**Table 3:** P-values from chi-squared test on the difference of log likelihoods.

		Full Model			
		Model 2	Model 3	Model 4	Model 5
	**Model 1**	4.397e-05***			
		(df = 14)			
	**Model 2**		0.086^+^	1.582e-13***	
**Null Model**			(df = 15)	(df = 16)	
	**Model 3**			7.220e-14***	
				(df = 16)	
	**Model 4**				0.035**
					(df = 16)

Significance Level

*** p<0.01

** p<0.05

^+^p<0.1. Rejecting the null hypothesis implies statistically significant improvements in model fit for the full model over that of the null model.

In the final model specification (Model 5), the total count of cells that burn zero times is off by less than 0.5% for the 25-year estimation period (1976–2000); the same can be said for cells that burn at least one time over this period. On the other hand, an estimation for the previous period (1951–1975) indicates that the number of pixels with at least one fire event is underestimated by approximately 1.25%, whereas the number of pixels with zero fires is overestimated by a similar amount. The remaining fire counts (i.e., areas that burned more than once) are over- or underestimated by less than 0.5% of the actual count for both the estimation period and the previous period.

### Model Interpretation

Within the final specification (Model 5), the fire count component is estimated by six variables: *cwdAave*, *aetAave*, *slope*, *DenMax25km*, *CampDist*, and *PublicLand*. These variables efficiently describe the total count of wildfires as well as the location of true zeroes (i.e., unfavorable conditions for fire). As expected, the non-linear fit of CWD and AET suggest that the number of fires initially increases with higher levels of AET or CWD but then decreases at very high levels of either (Fig A in S1 File). For AET, this indicates that transitioning from an area unsuitable for continuous vegetation growth (e.g., deserts) to more productive environments increases the number of expected fires. Finally, the number of fires declines at very high levels of AET as highly productive areas become too wet to burn frequently. A similar non-linear fit applies to the effects of neighboring housing density (*DenMax25km)*. In areas with a low adjacent housing density, the likelihood of fires increases rapidly with additional density (i.e., increased ignitions) up to some turning point, beyond which, the effects err towards increased levels of suppression or lack of combustible materials due to the built urban environment. Across all landscapes the number of fires increases with proximity to public and private campsites. Finally, the *PublicLand* variable indicates that, on average, there are more fires in public lands than on private lands throughout the state.

The zero-inflation component of the negative binomial model is comprised of *Ppall*, *Elev*, and *Lightning*. These variables determine the likelihood of observing a false zero. False zeros are comprised of unobserved fires, early suppressed fires, or areas of potential fires that have not yet burned. Looking at the example of the non-linear *Ppall* specification, it is evident that the likelihood of a false zero declines near populated places. Beyond a turning point around 20km, and with greater distance, the likelihood of a false zero increases. This indicates that fires close to population centers are more likely to be both observed and recorded, or potential fires ignited as actual fires. In contrast, at high distances from populated places more small fires go unobserved or ideal conditions unignited, creating more false zeroes. Looking at the effects of elevation, the non-linear specification indicates that increased elevation decreases the likelihood of false zeroes (small fires less often suppressed, greater likelihood of natural ignitions), but the degree of influence decreases rapidly in a logarithmic form. Higher lightning density also increases the likelihood of false zeroes as many small fires may be ignited but extinguish unobserved during storm events. Note that the coefficient for lightning in Model 5 is both large and has the inverse sign than in Model 1 (a model where human ignition and suppression were not included), indicating fewer false zeroes. This implies that ignoring human contributions to ignitions would strongly overstate the role of lightning as an ignition source.

### Historic Climate

Patterns of California’s fire activity encompass a broad gradient of environmental conditions, resources to burn, and ignition patterns. While the actual distribution of discrete fire events will differ from the modeled one (see Fig B in S1 File for actual fire count over 1976–2000), the modeled distributions reflect the likelihood of a discrete fire event occurring at a given location within a 25-year time period (Fig 2A). Model 5 estimates that each pixel will experience one fire event at most every 30.7 years, and every 377 years at the median; in contrast other areas are never expected to burn in a reasonable time frame. Areas currently with the shortest mFRI follow patterns of chaparral and oak woodlands due to their favorable wildfire conditions (Fig 2A). Areas with the longest mFRI that are expected to receive few if any fires (Fig 2A) include most of the cool and wet western edge of the Klamath Mountains, the high deserts east of the Sierra Nevada, the warm deserts of the southeast, and the isolated sagebrush steppe of the Modoc Plateau.

Comparing the mFRI from 1951–1975 and that from 1976–2000 allows us to visualize how climatic, weather, and human distributions changed California’s historic fire frequency (Fig 2B). Between the two periods we estimate a decline in mFRI (i.e., an increase in fire frequency) across areas of coastal influence in southwestern California by 1976–2000. Although this shift is small in absolute terms, it is large relative to the current mFRI of some locations. For instance, many fire prone areas in and around Los Angeles and San Bernardino National Forests experienced an estimated reduction in mFRI of 50%.

**Fig 3:**
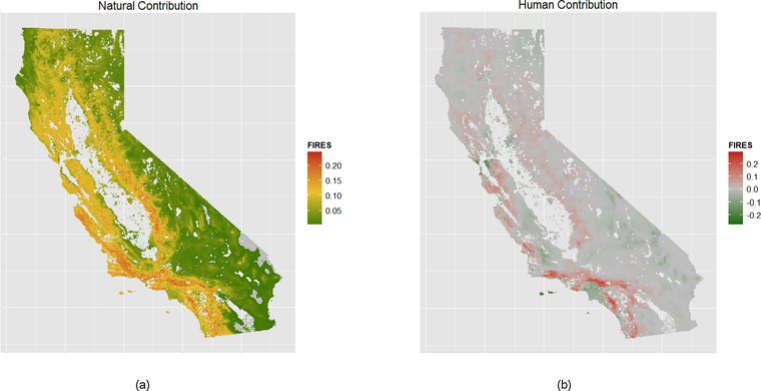
Contribution to 25 year expected fire count by natural and human factors. Contribution of natural (A) and human (B) variables, ceteris paribus, to the expected fire count predicted during the 1976–2000 period. Natural variables are defined as all variants of AET, CWD, lighting, elevation and slope. Human variables include distance to population centers, neighboring maximum housing density, distance to populated places, and distance to campsites.

As described in the methods section, we can map the influence of human and natural variables on the total fire count for the estimation 1976–2000 period (Fig 3). The influences of climate and terrain (Fig 3A) contribute the most to fire counts on the southern coast and in the southwestern Sierra, roughly tracing the current distribution of chaparral plant communities. Fig 3B maps the suppressive effect of the urban environment as well as the strong influence of humans in igniting fires in the wildland urban interface and neighboring public lands. Across all California, we find increases in the likelihood of fires up to approximately 10 kilometers from a population center, with little influence past 30 kilometers. But the intensity of fire response to anthropogenic influences varies by ecoregion, with the southwestern part of the state having the strongest human positive influence on fire counts. We can also see that the urban areas of central and southwest coasts have strong suppressive effects close to their centers.

### Future climate

**Fig 4:**
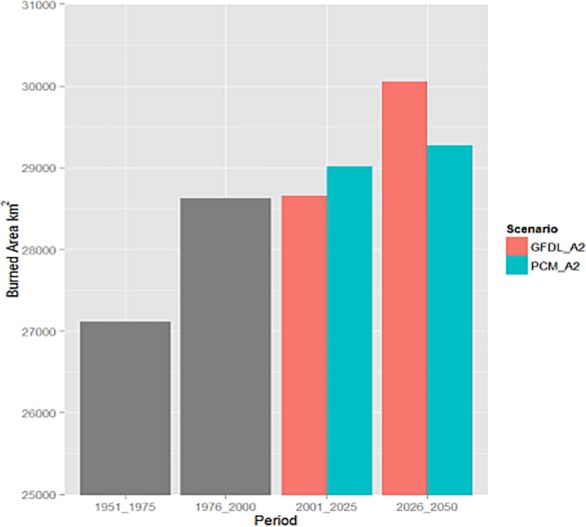
Area burned (Km^2^) by modeling period and climate model. Grey bars depict the area burned over the historical (1951–1975) and estimation (1976–2001) periods, whereas red and green bars depict the expected area burned into the future under the Geophysical Fluid Laboratory (GFDL) and Parallel Climate Model (PCM), respectively.

**Fig 5:**
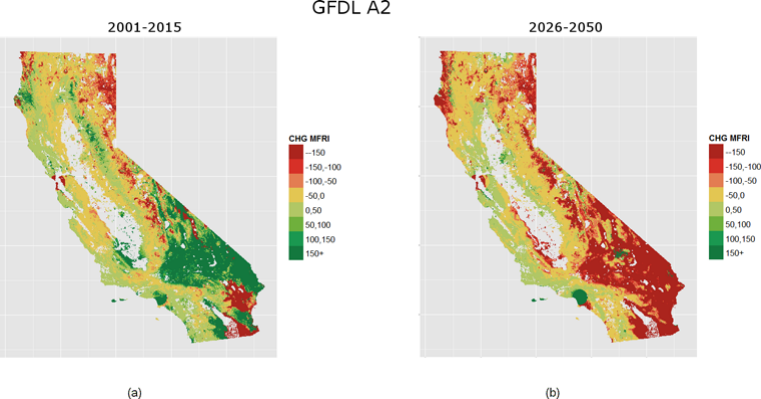
Change in mFRI by 2001–2025 and 2026–2050 under the GFDL climate scenario. Panel (A)depicts change in mFRI from the estimation period 1976–2000 to the 2001–2025 forecast period, whereas (B) show the expected change in mFRI by 2026–2050. Note that positive values indicate increasing mFRI or fewer fires, and the inverse for negative values.

**Fig 6:**
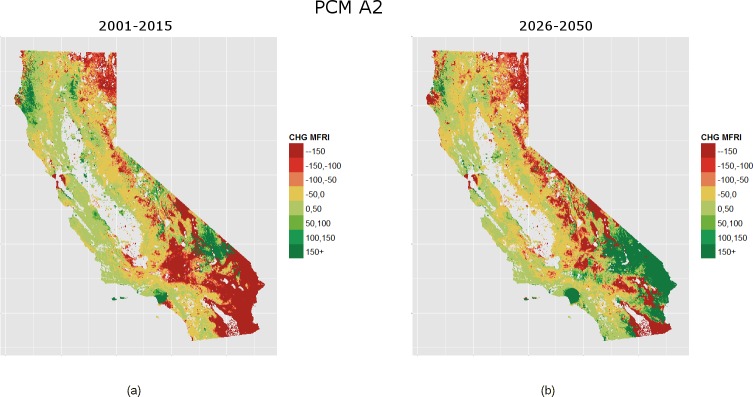
Change in MFRI 2001–2025, 2026–2050 PCM compared to 1976–2000. Change in MFRI for the PCM climate from the base period 1976–2000 to the first forecast period 2001–2025 (A), the change in MFRI from the base period 1976–2000 to the second forecast period 2026–2050 (B). Positive values indicate increasing MFRI or fewer fires, and the inverse for negative values.

**Fig 7:**
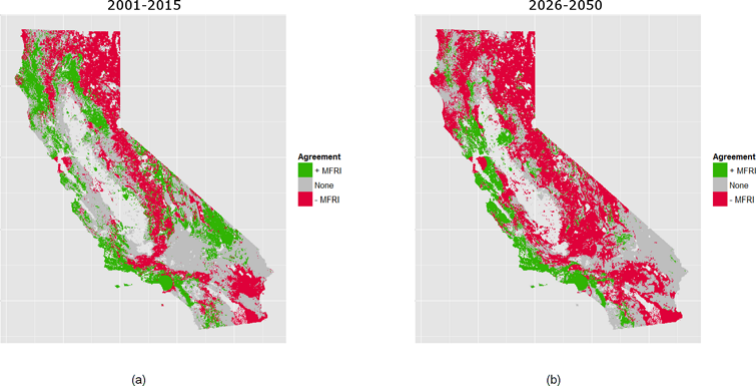
Model agreement for change in MFRI 2001–2025 and 2026–2050 compared to 1976–2000. Model agreement between the GFDL and PCM A2 scenarios for the (A) 2001–2025 and (B) 2026–2050 periods. Green indicates areas where model estimations from both GFDL and PCM models indicate increasing MFRI, or fewer fires. Red indicates areas where model estimations from both indicate a decreasing MFRI, or more fires. Grey indicates areas of disagreement.

Our analysis reveals that total area burned is expected to increase for both climates (PCM, GFDL) and time periods (2001–2025, 2026–2050) (Fig 4). We find that burned area is expected to increase by 2.2% and 5.0% by 2050 for the PCM and GFDL models, respectively. Shifts in climate and the built urban environment are reflected in 2001–2025 GFDL through an increase in the modeled number of years between fires for fuel-limited regions and a decrease in condition-limited areas (Fig 5A, Fig 2C). Under PCM climate projections the model forecasts a higher increase in the number of expected fires for the 2001–2025 period than GFDL over most of California (Fig 6A). This is especially true through much of the south and also the Sierra Nevada. Although the two climate models considered represent very different climate realizations their outcomes agree in many regions (Fig 7A).

In the second forecast period (2026–2050), GFDL simulations show higher fire activity than the PCM model (Fig 5B, Fig 6B). Such reversed trends in relation to the 2001–2025 period are due to greater moisture and fuel availability in the Sonoran and Mojave deserts under the GDFL scenario, drastically increasing the amount of fire-prone land. The GDFL model also estimates a reduction in the time between fires for many urban areas including Los Angeles, San Diego, and other coastal cities of the southwestern part and the Bay Area (Fig 5B). This reflects modeled increases in housing density throughout the WUI as well as modeled increases in moisture availability. On the contrary, due to declines in water availability, a reduction in the number of fire events applies to most urban areas under the PCM scenario, with increases in mFRI throughout the central and coastal south. The two climate models forecast a significant reduction in mFRI for the coastal influence zones of the northwestern part of the state, the higher elevations of the Sierra Nevada, and the Modoc Plateau. Smaller reductions in mFRI are forecasted for the Sierra foothills, and for much of the Klamath and Cascade Mountains. In contrast, the two models forecast much of the coastal influence zones of the central Coast to see moderate reductions in the number of fire events, with the exception of the Bay Area.

## Discussion

Our model is able to successfully replicate the distribution of observed fires (Fig 2A, Fig B in S1 File) as well as some of their spatiotemporal dynamics across California. For instance, model estimates capture the decreasing mFRI (increasing fire frequency) throughout coastal southern California and the Sierra Nevada between 1951 and 2000 [12,53] (Fig 2AB). These changes can be explained by a changing climate but also through the dynamics of human settlements particularly in areas of coastal influence (Fig 3AB). Future changes in climate and the built urban environment are expected to further shift, sometimes in dramatic ways, the spatial patterns of fire frequencies across California. Such heterogeneity depends on the interacting effects of climatic and human drivers on fire occurrence.

### Climatic Controls of Fire

The use of GFLD and PCM projections can be thought as contrasted future climatic bookends [9,46,54]. Model forecasts suggest substantial agreement about spatial and temporal variation in future fire activity in California by mid-21^st^ century, irrespective of specifications of future climate and human activities (Figs 5A–7B). For instance, the model suggests declining fire counts in coastal influence areas between approximately 33.5–39° latitude (Fig 6A and 6B). This is in line with recent findings of declining future fire probabilities for Mediterranean ecosystems under climate scenarios projecting warmer and drier conditions [13]. Our models also indicate that fire activity may decline for many of California’s central and north coastal zones by midcentury. However, our approach reveals high fire sensitivity to climate projections and thus future uncertainty for most of the coastal influence zones south of Los Angeles. In these locations diverging future climates show dramatically different fire outcomes with mFRI change between -150 to +100 years (Figs 5B and 6B). Given recent history and population density in these areas, even small shifts in mFRI in and around Los Angeles, San Diego, Del Mar, Encinitas and the Eastern side of the San Francisco Bay may be the most significant for human systems. These areas have experienced numerous and notoriously damaging fire events, such as the Cedar Fire in 2003.

Historically, many high elevation sites in California rarely burned because of long-lasting snowpack [55]. With greater evapotranspiration and declining levels of available moisture under both future climates examined, our model suggests dramatic decreases in the number of years between fires for many of these high elevation zones. Other studies point to an increase in the number of wildfires due to increased spring and summer temperatures, early snowmelt, and decreased precipitation [9,56,57]. Our analysis indicates that this may be especially true for the Sierra Nevada where a changing climate could increase the number of fires (with mFRI reducing between 50–150+ years) on the eastern edge (not seen in other models [9,58]) and to a slightly lesser degree (with mFRI reducing 0–100+ years) below the western edge. The difference between eastern and western edges may be an indication that the variables (i.e., AET and CWD) used here capture some of the complex interactions of drainage networks and edaphic properties of the area. At the other end of the spectrum, in locations that experience ideal weather conditions for wildfire but a lack of continuous fuel (e.g., semi-arid deserts), small increases in rainfall would increase fuels available to burn [59]. Our forecasts suggest this could be the case of the Sonoran or Mojave deserts which could see reductions in mFRI by 150+ or more years by 2050 under the GFDL climate (Fig 5B).

Despite the paramount role of water and energy balances variation for fire occurrence, we do not find evidence that inclusion of measures of inter-annual variability in climatic water deficit, precipitation, or temperature statistically improves model performance. This finding is similar to that of Parisien et al 2014 for controls on fire in the boreal forests of Canada [60]. However, the ability of our model to simulate the effects of variability on wildfire may be limited. Our model uses mean annual variation and does not account for specific sequences of dryer and wetter years, due to the long estimation period and challenges of GCMs in predicting such sequences [23]. Our ability to forecast the effects of variation is also limited by the GCMs’ reliance on the mean of many simulations, muting the effects of extreme events and intra/interannual variability [61].

Our finding of a moderate increases in the number of fires in the two forecast periods evaluated (2001–2025, 2026–2050) are in agreement with Lenihan et al [11], who use dynamic global vegetation models (DGVMs) to forecast relatively small increases (on the order of 4–7%) to burned area but with broad shifts in fire regime and plant community patterns. The similarities of our statistical approach with such process-based approaches suggest that AET and CWD are useful proxies for the determinants of both a shifting plant community as well as wildfire distribution [9,62].

### Anthropogenic influences on Fire

Many wildfire forecast models do not, or are unable to, include anthropogenic influences in a meaningful way [9,10,12,14]. For instance, many models drop urban areas from the sample while retaining other systems heavily influenced by humans (e.g. the WUI, federal and state parks) [9,12] or more generally omit anthropogenic variables [10,14]. As a result, much (or all) of the variability attributable to direct and indirect anthropogenic influences on fire is instead attributed to climatic ones. We find, however, that in many areas such as the WUI of Southern California, anthropogenic variables explain around half of the total fire count (Fig 3A and 3B). This is a classic case of omitted variable bias, whereby statistical models would attribute some part, or all, of the anthropogenic influences on variability to other variables retained in the model [63]. For example, comparing models 1 and 5 demonstrates that omitting anthropogenic influences significantly overstates the role of lightning as an ignition source, and increases the size coefficients for climate-relevant variables (*aetAave* and *cwdAave*) by 24% on average. Therefore, our findings evidence that the failure to include anthropogenic effects in future fire estimates may be significantly overstating the response of wildfire to climatic change alone.

Although humans can have diverse effects on fire regimes (Fig 1), they alter them through three dominant mechanisms: increased ignitions, fire management practices, and the modification of land cover [21]. Our modeling approach suggests that there is a strong anthropogenic influence on mFRI throughout California, with fire activity increasing in public lands, especially those surrounding urban areas, alongside of strong forces promoting fire suppression through firefighting and the physical properties of a dense urban environment. Overall, our results indicate that spatial heterogeneity in fire’s response to anthropogenic influences between ecoregions and between urban and rural communities is complex [1] (Fig 3B). Our model is able to simulate that suppression is less effective in drier regions subject to Santa Ana winds than in wetter climes such as in the San Francisco Bay Area, which are also subject to dry but less frequent easterly winds. Our model also captures changes in observed fire frequency that is likely due to changes in anthropogenic forces. For example, the model estimates indicate that many fire prone areas in and around San Bernardino National Forests in southern California experienced a reduction in mFRI of 50% (more frequent fire) from 1951–1975 to 1976–2000. These estimated changes in anthropogenic influence (seen in Fig 3B) match the expected influence of higher-density housing that occurred in tandem with invasive species, and with more favorable climatic conditions for fire [53]. Finally, our findings also suggest, on average, higher increases in the number of fires across public lands. This may be reflecting differences between private and public land management associated with the level of suppression and mitigation effort exerted in both landscapes. Additionally, the significant influence of distance to public campsites in our model highlights that even low levels of human activity (as may be the case in many public lands) can have a measurable effect on the likelihood of fires in wilderness areas [64].

### Modeling Assumptions & Limitations

As with any forecasting model, a number of assumptions are made to facilitate estimation. First and foremost, we assume that the CMIP3 PCM and GFDL A2 scenarios act as a representative climate envelope that brackets the possible range of future conditions for California (see Forecasting Considerations section). Because the training data are cross-sectional (covering one-time period; 1976–2000), our approach also assumes that major vegetation patterns and their response to wildfire should follow the 25-year climate norms bounded by these models. Problems associated with this assumption may be partially reconciled by the broad range of ecosystems and climates in California. That is, given the existence of multiple observations of vegetation types in various stages of succession over the calibration period (1976–2000), for any given level of AET and CWD, the model will assign an average fire response that encompasses such variation. The results therefore should be relatively conservative, fitting the mean fire response to an ensemble of successional stages and vegetation types, rather than over-fitting to a deterministic equilibrium ecosystem type. Note that wildfire can also respond to changes in vegetation through non-native introductions, such as the invasive grasses of the Mojave [65], or through invasive pathogens such as sudden oak death [66]. Such processes are unlikely to be captured in our approach. Additionally, we do not include the effects of fuel treatment, which have been shown to be effective in some ecosystems [7]. Although the extent of these efforts are relatively limited, these effects should be included in future studies. The influence of severe weather and extreme events will be masked by both the climate models as well as through the use of 25-year norms in this analysis. The measure of wildfire counts used in this study, like most historical data, should be considered incomplete particularly before 1980; it is however the most comprehensive spatial data set for California. The use of a zero-inflated model, whereby we partially control for the additional ‘false zeros’ due to omission from the data, ameliorates this issue. Additionally, our approach assumes that the relationships between fire and climatic and anthropogenic variables (through fixed coefficients and shape parameter from the negative binomial model) are fixed over time. Therefore, any non-linear responses not observed in the sample period cannot be predicted for future periods. Additionally, although the use of a Negative Binomial distribution is more flexible than the Poisson distribution, the shape of the distribution is assumed to be fixed over time. This implies roughly that ratio between cells with low and high burn frequency remains fixed into the future. Finally, we assume that the intensity of lightning strikes is fixed over time. However, given the relatively small role of natural ignitions in California, this should not greatly influence model outputs.

## Conclusions

California occupies an unusually broad climate space, spanning alpine zones, temperate rainforest, Mediterranean ecosystems, and semi-arid deserts. California is also the most populous state in the U.S., with large communities developing in and around fire-prone landscapes. As these communities develop over time they will influence the balance between increasing and decreasing the likelihood of wildfire, reinforcing or mitigating the effects of climate change. Such influences extend beyond the reach of simple patterns of anthropogenic ignition and suppression, to include factors like public/private land management practices, introduction of invasive species, and the manipulation of local and regional hydrology. These facts, coupled with the strong evolutionary influence of fire on plant traits and community composition [67,68] make understanding fire regime shifts in response to climate and the built environment critical to maintaining biodiversity and minimizing risks to human systems. The importance of both anthropogenic and biophysical variables documented in this study emphasizes the need to incorporate a broad suite of influences in future projections of this essential ecological disturbance. The failure to include these critical anthropogenic effects in models will overstate the importance of climatic variables on wildfire.

## Supporting Information

S1 File Fig A. Nonlinear regression curves for selected variables. Nonlinear coefficient estimates traces the effect of key coefficients from the final model (5) in Table 2. Fig B. Difference between actual and predicted % of total fires for 1976–2000 and 1951–2000 estimates. Difference in the percentage of total pixels in each fire count category for 1976–2000 (top panel) and 1951–2000 (bottom panel). Values above zero indicate overestimates in the number of pixels for any given fire count class. The top panel presents each of the 30 individual model runs (blue) and estimates from the mean model (red) for the estimation 1976–2000 period. The lower panel presents the estimates from the 1951–1975 period for the mean model (red). Fig C. Actual wildfire count 1976–2000. Total count of wildfires for the 1976–2000 period as recorded by the FRAP fire record.
(DOCX)
