# Supplementary material for: Incorporating Anthropogenic Influences into Fire Probability Models: Effects of Human Activity and Climate Change on Fire Activity in California
Source: PLoS One. 2016 Apr 28;11(4):e0153589. doi: 10.1371/journal.pone.0153589 (PMC4849771; doi:10.1371/journal.pone.0153589)
Supplement: S1 File — Fig A. Nonlinear regression curves for selected variables. Nonlinear coefficient estimates traces the effect of key coefficients from the final model (5) in Table 2. Fig B. Difference between actual and predicted % of total fires for 1976–2000 and 1951–2000 estimates. Difference in the percentage of total pixels in each fire count category for 1976–2000 (top panel) and 1951–2000 (bottom panel). Values above zero indicate overestimates in the number of pixels for any given fire count class. The top panel presents each of the 30 individual model runs (blue) and estimates from the mean model (red) for the estimation 1976–2000 period. The lower panel presents the estimates from the 1951–1975 period for the mean model (red). Fig C. Actual wildfire count 1976–2000. Total count of wildfires for the 1976–2000 period as recorded by the FRAP fire record. (DOCX) [file pone.0153589.s001.docx]

## S1. Supporting Information

### Zero Inflated Negative Binomial Models

Only a handful of statistical methodologies are designed to handle these nuances of the data generation processes. One of them, the zero inflated negative binomial model (ZINB) is designed to handle both count data and the existence of ‘true’ and ‘false’ zeroes. The following section attempts to describe this rather sophisticated technique using simplified mathematics and language, and is largely adapted from Zuur’s excellent treatment in statistics for biology and health [1].

Let Pr(Y) be the probability that a site *i* has a wildfire. The probability of observing zero fire counts is:

$$\left( 1 \right) \Pr(Y_{i}=0) =Pr (False Zeros)+(1-Pr (False Zeros) ) \times Pr (Zero in count Process)$$

Equation (1) indicates that zeroes are generated from two sources, ‘false zero’ are generated in the zero mass or zero-inflation component, and ‘true zeroes’ are generated in the count component. Here the probability of a ‘true zero’ is the joint probability that it is both not a ‘false zero’ and is a zero in the count process. We essentially assume that there are two groups of zeroes, as discussed above: ‘false zeroes’ (unobserved/recorded, or suppressed fires) and ‘true zeroes’ (no actual fires). As such, the probability of Y_i_ being a false zero is binomially distributed with the probability
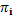

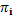
 and the probability of Y_i_ not being a false zero is
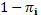
${1-\pi}_{i}$. We can therefore rewrite equation (1) as:

$$\left( 2 \right) \Pr(Y_{i}=0) =\pi_{i}+({1-\pi}_{i})\times Pr (Zero in count Process)$$

The remaining component is the probability for site *i* that a zero is derived from the count component. Here we assume, after testing, that our count process is best modeled with a negative binomial distribution. We are therefore assuming that the data is over dispersed, or the sample variance is greater than the sample mean. With the probability of $Y_{i}=0$ taking the form of:

$$\left( 3 \right) \Pr(Y_{i}=0) =\pi_{i}+({1-\pi}_{i})\times{(\frac{\theta}{\mu_{i}+\theta})}^{\theta}$$

Where
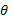
$\theta$ is the shape parameter and
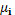

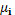
 is the expected value. Now that we know the probability of $Y_{i}=0$, we can solve for the probability of $Y_{i}>0$. Completing the probability distribution:

$$\left( 4 \right) \Pr(Y_{i}=k) = ({1-\pi}_{i})\times f_{\mathrm{NB}}(Y), k=1,2,\ldots,$$

$${where: f}_{\mathrm{NB}}(Y)=\frac{\Gamma(k+\theta)}{\Gamma(\theta)\times k!}\times{(\frac{\mu_{i}^{k}\theta^{\theta}}{{(\mu_{i}+\theta)}^{k+\theta}})}^{k} , k=1,2,\ldots,$$

We now have the complete probability distribution where we have defined the probability of zero and non-zero events $\Pr(Y_{i}=0)$ and $\Pr(Y_{i}=k)$, with expected values
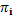
$\pi_{i}$ and $\mu_{i},$ and $\Gamma$ as the gamma function and
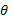
$\theta$ as the shape parameter, which allows us to adapt the shape and scale of the traditional Poisson distribution. We can then model the mean values of $\pi_{i}$ and $\mu_{i}$ by introducing two sets of covariates Z and X. For example, the average number of fires may depend on the length of the fire season and the number of ignitions, and the probability of a false zero may depend on the ability to observe the fire and the distance from populated places. Here we estimate the probability of a false zero $\pi_{i}$ with a logistic regression; to simplify the discussion we show $\mu_{i}$ estimated as a Poisson distribution [2].

$${{\left( 5 \right) \pi}_{i}=\frac{e^{\alpha+\beta_{1}X_{i2}+\beta_{2}X_{i2}}}{1+e^{\alpha+\beta_{1}X_{i2}+\beta_{2}X_{i2}}} \mu}_{i}= e^{\alpha+\delta_{1}Z_{i2}+\delta_{2}Z_{i2}}$$

Here ${\alpha, \beta}_{i}$ and $\delta_{i}$ are estimated regression coefficients. In the model $\mu_{i}$ follows a negative binomial distribution and is estimated in conjunction with the estimation of $\theta$, the negative binomial shape parameter. These parameters are then estimated by maximum likelihood.

### Supplemental Figures

**A Fig. Nonlinear regression curves for selected variables.**


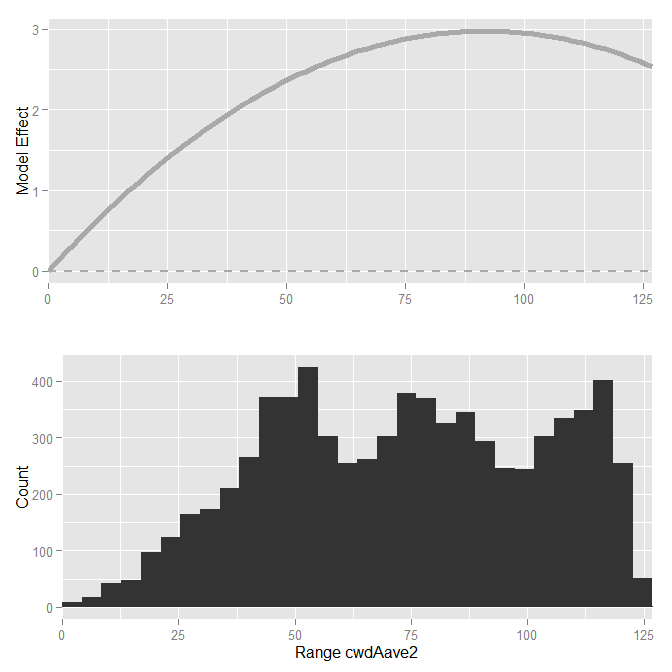

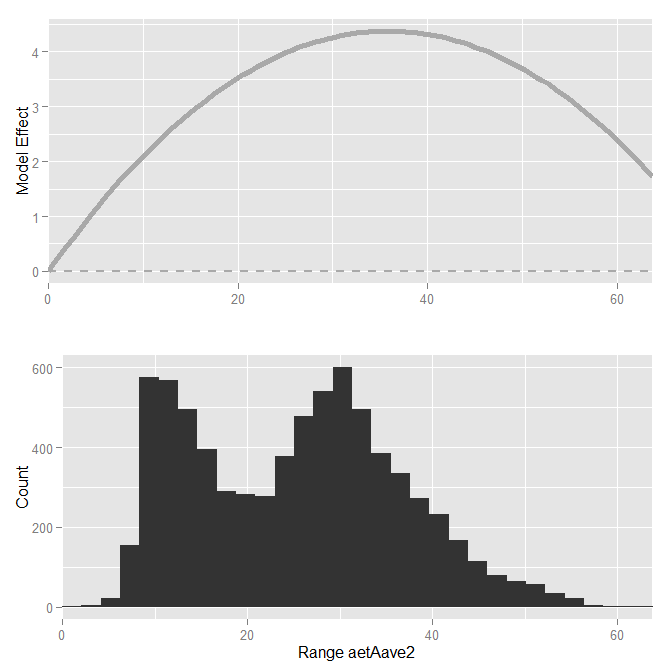

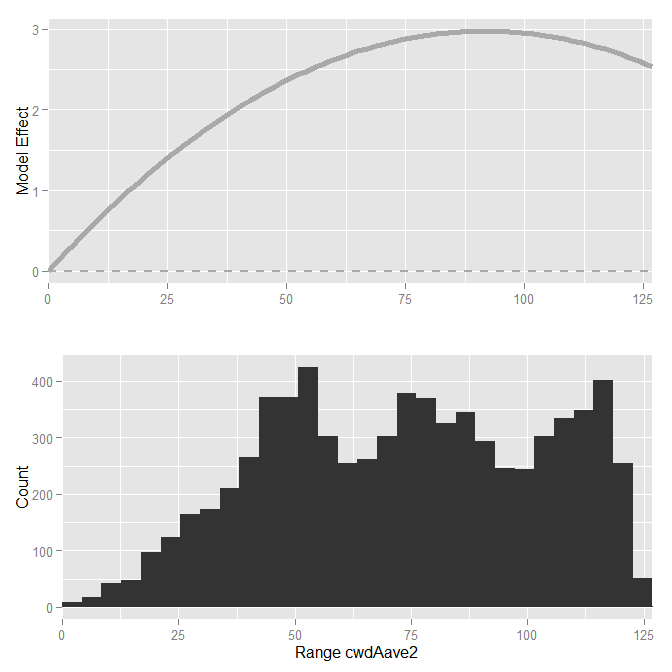

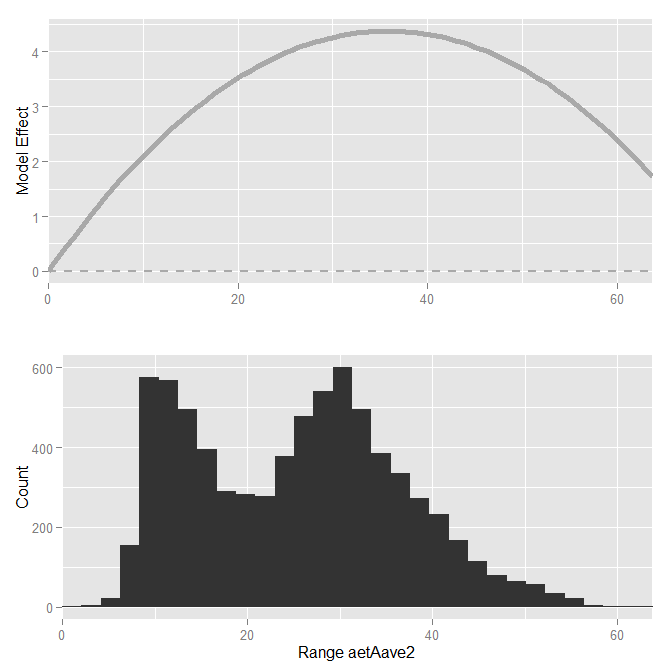


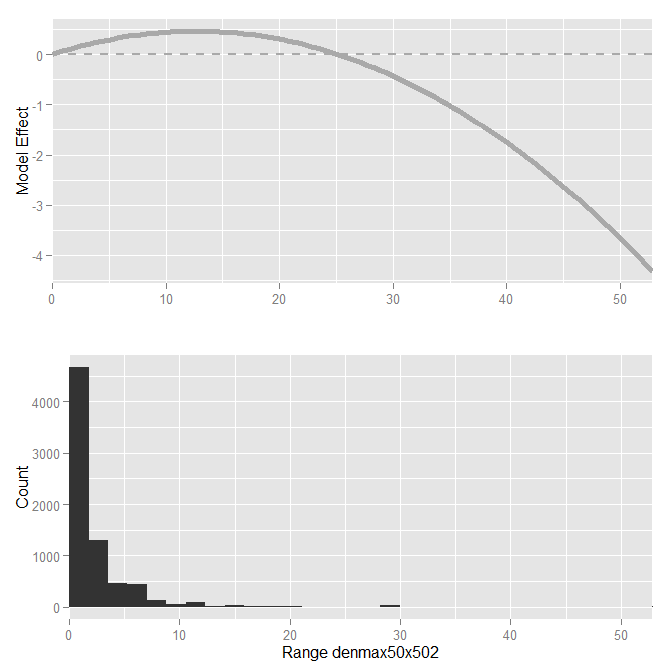

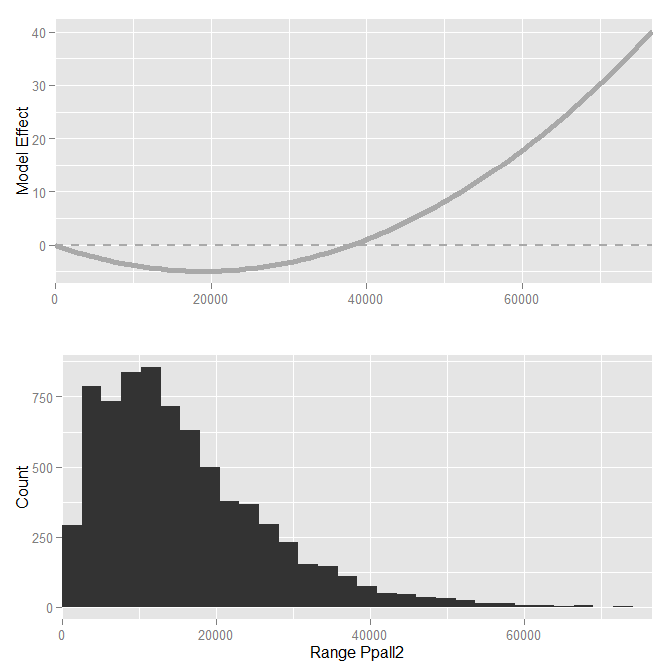

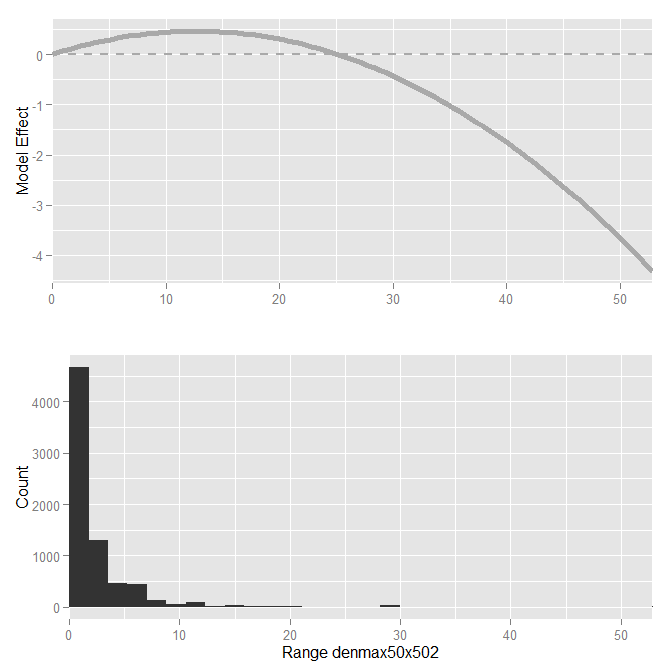

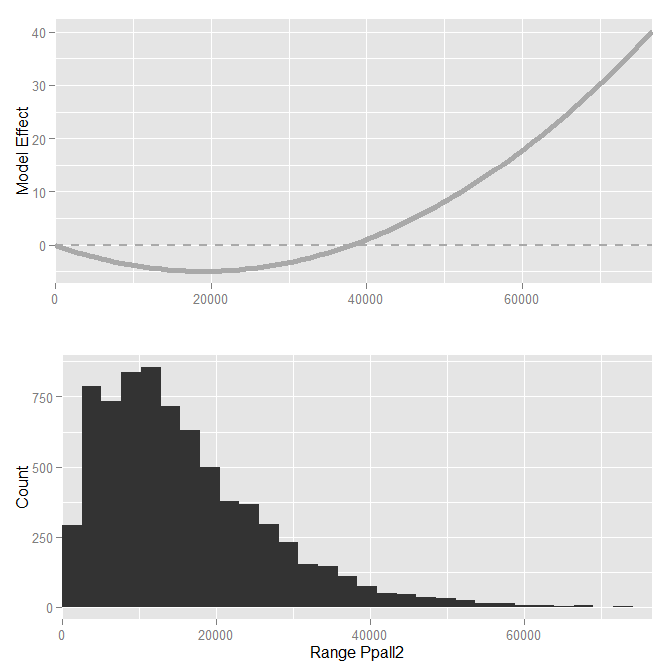


Nonlinear coefficient estimates traces the effect of key coefficients from the final model (5) in Table 2.

**B Fig. Difference between actual and predicted % of total fires for 1976-2000 and 1951-2000 estimates.**


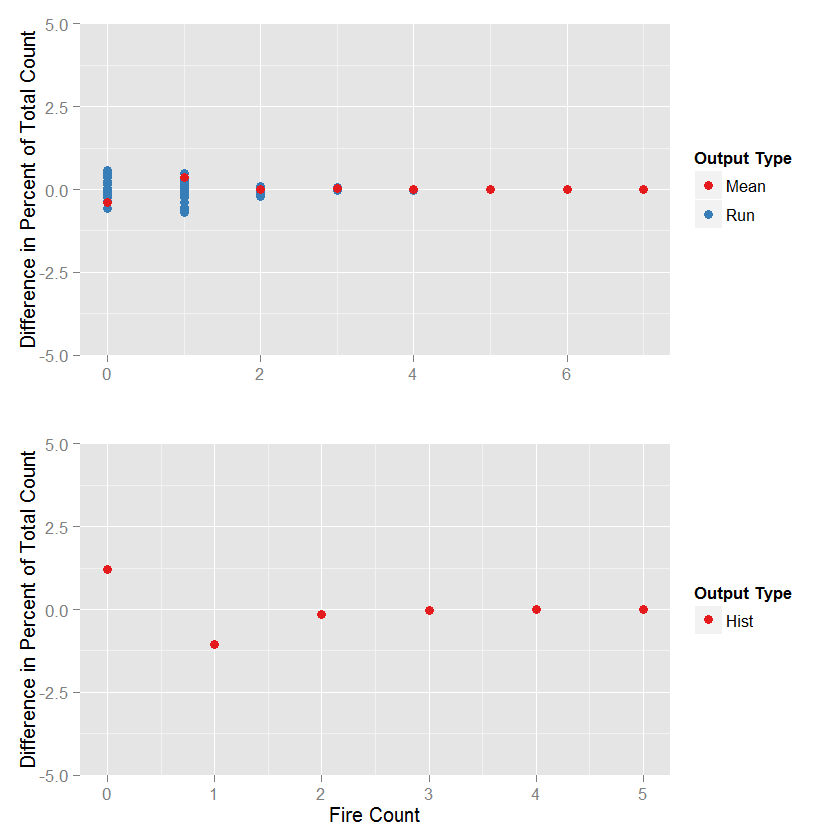


Difference in the percentage of total pixels in each fire count category for 1976-2000 (top panel) and 1951-2000 (bottom panel). Values above zero indicate overestimates in the number of pixels for any given fire count class. The top panel presents each of the 30 individual model runs (blue) and estimates from the mean model (red) for the estimation 1976-2000 period. The lower panel presents the estimates from the 1951-1975 period for the mean model (red).

**C Fig. Actual wildfire count 1976-2000.**


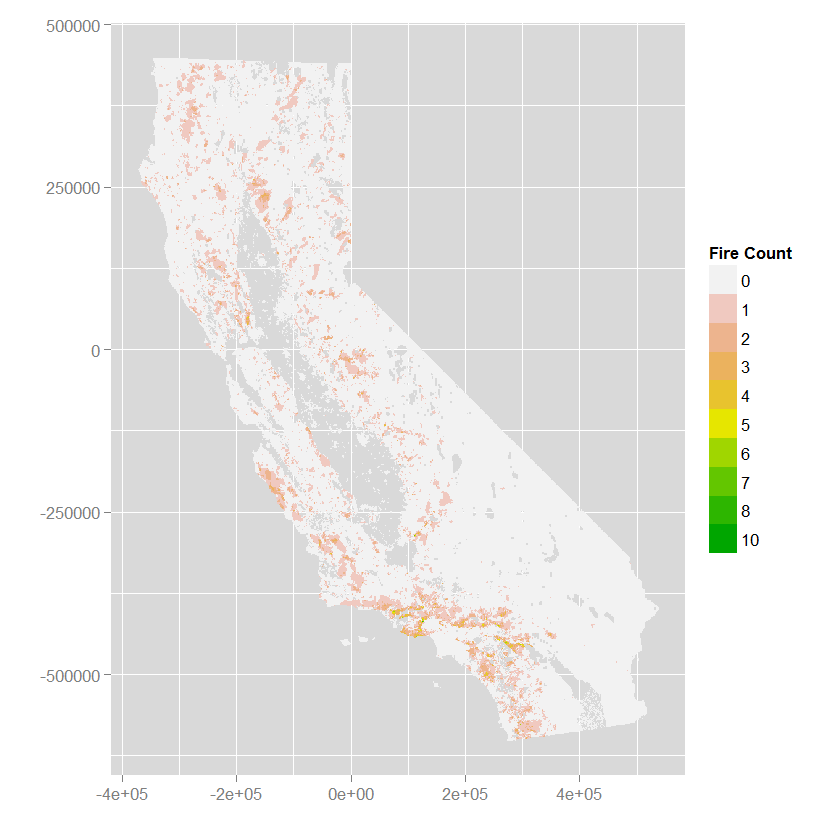


Total count of wildfires for the 1976-2000 period as recorded by the FRAP fire record.

## Works Cited

1. Zuur A, Leno EN, Walker N, Saveliev AA, Smith GM. Mixed Effects Models and Extensions in Ecology with R. New York, NY: Springer; 2009.

2. Loeys T, Moerkerke B, De Smet O, Buysse A. The analysis of zero‐inflated count data: Beyond zero‐inflated Poisson regression. Br J Math Stat Psychol. 2012;65: 163–180.
